# Closing Editorial: New insights and reflections on the science of selection and recruitment

**DOI:** 10.15694/mep.2018.0000287.1

**Published:** 2018-12-18

**Authors:** Fiona Patterson, Barbara Griffin, Mark Hanson

**Affiliations:** 1 Work Psychology Group; 2 Macquarie University; 3 University of Toronto

**Keywords:** selection, recruitment, diversity, technology, widening access

## Abstract

This article was migrated. The article was marked as recommended.

In this closing editorial we reflect on key topics presented in this special issue on selection and recruitment, with a view to identifying gaps in the literature, and exploring where next. Four key themes have emerged including; (1) the impact of using new technologies in selection and recruitment; (2) addressing social accountability, diversity and fairness issues in selection; (3) increased emphasis on non-academic personal attributes in selection, and (4) attraction and recruitment in postgraduate recruitment. The implications of findings from this collection of studies and opinion pieces are discussed in relation to future research, policy and practice.

## Introduction

This special issue provided a platform to present the latest research, opinion pieces, case studies, and commentaries on current and future issues in selection and recruitment in medical education. New insights into the state of the science within medicine and other healthcare professions are offered, and in this closing editorial we reflect on key topics, with a view to identifying gaps in the literature, and exploring where next.

The various papers in this issue clearly demonstrate that research in this area is moving beyond focusing on evaluating the quality of selection methods alone to consider more complex topics, such as the political landscape of selection and tensions between stakeholders, new selection criteria and outcomes of interest, the use of emerging technologies, and case studies of practical interventions of how to deal with social accountability and diversity issues.

Most of the previous literature has tended to focus on evaluations of medical school admissions and it is refreshing to see an increasing focus on postgraduate recruitment ( Barajaz et al, 2018), dealing more with attraction issues ( Cunliffe et al, 2018), especially in those specialties where there are workforce shortages. In addition, new topics are emerging including issues regarding self-selection and analysis of person-organisation fit as part of recruitment ( Rothstein et al, 2018) and using selection tools for educational purposes in addition to making selection decisions ( Gardner and Dunkin, 2018).

In overview, we identify four emergent themes (amongst others) including; (1) the impact of using new technologies in selection and recruitment; (2) addressing social accountability, diversity and fairness issues in selection; (3) increased emphasis on non-academic personal attributes in selection, and (4) attraction and recruitment in postgraduate recruitment. Here, we briefly summarise each of themes in relation to the papers published in this issue.

## Theme 1. New technologies (machine learning, social media, virtual approaches) will have a major influence on the design of selection approaches and recruitment practices in future

In exploring the mechanics of how researchers can design and validate different selection tools and systems, Paton and Tiffin (2018) offer important new insights in how artificial intelligence (AI) and machine learning can be applied to selection, demonstrating the potential utility using case examples. As the reviewers highlight, the approach offers the possibility for non-linear combinations of data to be analysed, going beyond what researchers have typically treated as linear data, which do not adequately account for what is usually complex data. The reviewers also highlight caution regarding the ‘black box’ issue of machine learning decision making, where results are not readily interpretable for researchers, let alone practitioners. Regarding fairness, how would some candidates (and indeed other stakeholders) feel about being rejected based on an algorithm that cannot be easily explained against a person specification and the espoused selection criteria? Candidates expect meaningful feedback which is also related to their propensity for legal case initiation ( Patterson & Zibarras, 2011), and simply reporting “ *the computer says ‘No’”*, is unlikely to be satisfactory. That said, the reviewers also highlight that such an approach however could be very helpful for formative purposes to promote early educational interventions if a student/trainee is at more risk of falling into difficulty. All said, the use of AI in selection promises to be fertile ground for further research.

Following the theme of new technology, several papers in this issue demonstrate how social media can be especially advantageous for attraction and outreach in recruitment (e.g. use of videos on Twitter for psychiatry recruitment ( Cunliffe et al, 2018) and use of live web chats for school leavers; ( Garrud et al, 2018), in addition to offering more efficient approaches to delivering selection methods (e.g. piloting asynchronous MMIs in medical school admissions; Zibarras et al, 2018). In general, the results are positive and further research is required to understand candidate perceptions to the increased use of new technology in selection. For all stakeholders, researchers are encouraged to explore the extent to which enhanced technology in selection and recruitment can improve efficiency and effectiveness in the long term.

## Theme 2. New insights in how to address promoting diversity and improving widening access

Fairness and diversity continue to be critically important issues in selection and recruitment practices and several papers in this issue explore new approaches in terms of attracting, recruiting and supporting under-represented groups into medical school, through to specialty training and into practice.

In reviewing issues regarding socio-economic status (SES), Fortin et al (2018) highlight how the financial demands of preparing and completing aptitude tests for entry into medical schools in Canada contributes to the current diversity challenges, where most respondents to their survey agreed that the MCAT posed a financial hardship, especially for those from low income families. So how do schools and society in general provide a level playing field whilst addressing the interests of social accountability?

In a provocative opinion piece, Nicholson (2018) highlights that these issues are difficult to resolve since widening participation remains a contested field without consensus between stakeholders about best practice within selection and recruitment. She argues that a fundamental change in thinking is required to truly diversify the healthcare workforce and that a major limitation is over-reliance on prior academic attainment or ‘academic readiness’ as the main selection criteria. Since those from lower socio-economic backgrounds tend to have less access to good quality education they are at an immediate disadvantage (and indeed, experience less access to admissions test preparation activities).

All authors agree that further research is urgently required to help schools gather evidence internationally as to ‘what works’ in making the significant changes necessary to facilitate selecting a more diverse medical workforce. An important part of this equation is in ensuring access to good quality outcome data (e.g. retention, specialty choice, in-training performance, licensure, and ultimately, health outcomes) to allow longitudinal tracking to evidence the impact of any policy changes.

One approach is offered by our Dutch colleagues ( Wouters et al, 2018), who review the research evidence on the lottery system that was used for many years for medical school admissions in the Netherlands to promote diversity in the student intake. Considering the perspectives of various stakeholder groups (i.e. applicants, medical schools and society), Wouters et al conclude that both the lottery and selection approaches each yield a combination of advantages and disadvantages, such that none of the currently available admissions strategies completely fulfils stakeholders’ needs in this country, and hence why the field remains contested. They also highlight the tension between institutions’ pursuit of prestige versus society’s needs for a diverse workforce. Future research could usefully explore approaches to resolving such tensions, without which practical solutions to adequately address widening access and diversity will remain elusive.

There has been much debate regarding how to design selection methods to address diversity (e.g. see Parsons et al (2018) study of using traditional interviews versus MMIs in medical school admissions in Newfoundland, Canada). Less research has focused on practical solutions involving new technology in recruitment and in this special issue there are some good examples ( Garrud et al, 2018). That said, there remain many hurdles to jump, not least in exploring which markers are the most effective in targeting outreach programmes (Satarnia et al, 2018).

Regarding gender and diversity, Traynor et al (2018) provide a UK-based research study exploring issues regarding fairness within the nursing profession, where less than 11% of registered nurses are men. The introduction of a new MMI as part of the selection process was perceived to be fair by men, but authors remind us that, given prior research showing females outperform males on MMIs, careful attention is required in the design of stations so as not to unfairly advantage any group of candidates. Fairness for all applicants remains an important consideration in the design of selection methods and systems.

In summary, there are many dimensions to consider in establishing how best to deal with diversity issues, ranging from the micro-level (e.g. MMI station design, markers to be used in identifying pockets of disadvantage) through to the macro-level (e.g. resolving competing tensions in stakeholder needs and wants).

## Theme 3. An increased emphasis on non-academic selection criteria and whole-person approaches for the future

A key theme in this issue is in designing selection methods and systems that account for a range of personal attributes and values important for anyone entering a career within healthcare. For example, Yingling et al (2018) explore holistic approaches to selection and suggest that professional identity formation can provide new ways to conceptualise students’ readiness for medical school. As the discipline of medicine is developing rapidly and the demand for good quality healthcare is increasing globally, selection and recruitment practices (alongside educational programmes and interventions) must address these challenges. In order to cope effectively with such demands, Fink et al (2018) explore the concept of ‘grit’ in residency performance, described as a trait possessed by individuals who demonstrate perseverance toward a goal despite being confronted by significant obstacles and distractions. Although results were mixed and inconclusive, it is notable that researchers are now exploring a broader range of intra- and inter-personal attributes in selection.

## Theme 4. Going beyond selection approaches to considering issues regarding attraction, especially in postgraduate recruitment

Much previous research has tended to focus solely on selection issues rather than tackling the broader aspects of attraction and recruitment. However, this topic is reflected in several papers in this special issue, especially for postgraduate residency selection (e.g. Barajaz et al, 2018; Parsons et al, 2018) where many specialties face significant workforce shortages (e.g. Psychiatry; Cunliffe et al, 2018). The problems are compounded in certain contexts where workforce shortages can be especially acute in remote and rural locations (e.g. Parsons et al, 2018). Further research here, drawing on insights from the broader selection literature on attraction and recruitment in professions outside of medical education, would be very welcome.

## Future directions for research and implications for practice

It is clear from the number and variety of submissions to this special issue that the science of selection and recruitment in healthcare is a growing topic of interest, new insights have emerged, and yet there is much still to be explored.

The use of technology in selection is an exciting and fruitful avenue for research from a variety of vantage points, not just in the delivery of selection tools, but also in influencing data analysis approaches to more adequately account for the complexity of the issues. In the corporate world there have been many studies of the use of technology in selection and further research is required in selection for medical education, which has so far been relatively untouched.

Following a recently published Ottawa consensus statement and recommendations on selection and recruitment ( Patterson et al, 2018), many of the papers in this issue have begun to address these recommendations by, (i) paying greater attention to the interaction between selection methods and selection philosophy and policy making, and (ii) using new evaluation approaches both in data analysis and exploring new attributes and outcome markers. Much is still to be done however, and future research could focus on inter-disciplinary research to also include the tacit knowledge of those responsible for delivering selection ( Lee, 2018), involving a more diverse group of stakeholder perspectives, including patients ( Lombard et al, 2018). In turn, there is a need to use new and different theoretical frameworks to provide better quality evidence to help educate those directly involved in selection and recruitment to navigate the complexity of the issues.

In the past decade, robust evidence for the quality of different selection methods has materialised. To what extent however has this translated into policy in practice? We argue that there continues to be a major gap between research and practice and so understanding how to best bridge this gap is an area that needs serious attention.

Much of the extant literature continues to be at the micro-level of analysis yet we urge researchers to also consider macro-level analyses to provide a more rounded picture of the issues. Practically however, we acknowledge that conducting good quality, longitudinal, multi-site, multi-source, multi-method, macro-level studies is clearly difficult for a variety of reasons. Further dialogue with a broad range of stakeholders (to include policy-makers and fund-holders) should aim to systematically unpick why this is the case and suggest ways in which this can be overcome (or not).

Almost all papers in this issue focus on studies arising from North America, Europe and Australasia. García-Estañ (2018) provides a useful overview of medical selection in Spain, which has rarely featured in previous research. However, since selection policy and practice are shaped by a range ofsocial, political and culturalforces, how can we encourage researchers from many other parts of the globe to join the debate? Similarly, we urge researchers to explore cross-country comparisons to offer new insights. A good example is the review of the lottery system for medical school admissions in the Netherlands presented in this issue. How are recruitment philosophies and policies formulated in other parts of the globe?

In exploring philosophies for selection, how can the conflict be resolved between selections systems based on a meritocracy versus those designed to address workforce shortages in some specialties and communities? The jury is still out and future international research collaborations may offer some promise in unpicking the issues (see INReSH; International research Network for Researchers in Selection into Healthcare https://www.medschools.ac.uk/our-work/selection/inresh, a new research network designed to address the strategic and societal challenges that shape selection practices, and how this in turn determines avenues of future research).

International research collaborations are one step forward but bridging the academic/practitioner divide in selection policy in future will require engagement from a broader range of stakeholder perspectives. The new Ottawa consensus statement speaks mostly to a research agenda ( Patterson, 2018) and much work is needed to translate findings into practical guidance. In this issue, Lee (2018) provides some important insights from an admissions officer perspective, a voice seldom heard in the research literature, highlighting the need for closer connections between academics and selection committees to engage in dialogue about knowledge translation into practice. Lee (2018) argues that admissions officers should play a more prominent role in facilitating developments in the field by offering knowledge, operational experiences and networks. Future research should consider which other stakeholder groups and audiences could offer the much-needed support in translating research into policy and practice.

There is over a century’s worth of international research literature exploring selection and recruitment across many different professions but historically less attention has centred on selection into the healthcare professions. This picture is changing rapidly, and it is clear that selection into medical education presents some unique challenges. We would like to thank all the contributors to this special issue in taking the dialogue forward with a view to supporting recruiters, researchers, educators and students in understanding those challenges to further inform future policy and practice.

## Take Home Messages

• Research in selection and recruitment in medical education is moving beyond evaluations of selection methods to consider more complex topics, including the political landscape of selection and tensions between stakeholders, new selection criteria and outcomes of interest, and practical interventions of how to deal with social accountability and diversity issues.
• New technologies (machine learning, social media, virtual approaches) will have a major influence on the design of selection approaches and recruitment practices in future.
• In addressing diversity and widening access issues in recruitment, none of the currently available admissions strategies completely fulfils differing stakeholders’ needs, and hence why the field remains contested.
• There continues to be a major gap between research and practice in selection and recruitment and understanding how to best bridge this gap is urgently required.
• International collaborations between researchers to also include a broader range of stakeholder groups, including policy-makers and fund-holders, are likely to yield new insights in how to translate the extant research into practical guidance.

## Notes On Contributors

Professor Fiona Patterson is a founding Director of Work Psychology Group, a research-led consulting practice with specialist expertise in selection. She is a Visiting Researcher at the Universities of Cambridge, London, Nottingham and Aberdeen. She Co-Chairs an international research network for selection for the healthcare professions (INReSH) with contributors from around the globe. She led the 2018 Ottawa consensus statement on selection and recruitment in healthcare hosted by AMEE. ORCID https://orcid.org/0000-0002-1031-130X

Professor Barbara Griffin is a member of the Department of Psychology at Macquarie University and an endorsed organisational psychologist. She has led the development of selection processes for both undergraduate and postgraduate medical programs in Australia and consults to industry and specialist medical colleges on selection issues. ORCID https://orcid.org/0000-0002-3597-7351

Dr. Mark D. Hanson is a Psychiatrist at the Hospital for Sick Children and Professor, Department of Psychiatry, Faculty of Medicine, University of Toronto, Canada. Previous medical education positions held include Associate Dean/Director Admissions and Student Financial Aid, Undergraduate Medicine, Faculty of Medicine, University of Toronto. Admissions scholarship focuses upon issues of social accountability, admissions interviewing and file review methods. ORCID https://orcid.org/0000-0002-0820-4521

## Declarations

Fiona Patterson is a Director in Work Psychology Group, a research consulting practice which receives funding from Health Education England, UKCAT and other educational institutions to design and evaluate a range of selection methods and systems. Fiona is a guest Theme Editor for the theme of Selection and Recruitment in Medical Education in AMEE MedEdPublish.Barbara Griffin is a guest Theme Editor for the theme of Selection and Recruitment in Medical Education in AMEE MedEdPublish.Mark D. Hanson holds an Associated Medical Services (AMS) Phoenix Fellowship Award to examine a New Health Professions Admission Model Grounded in Patient Defined Application Attributes, Processes and Policies. Mark is a guest Theme Editor for the theme of Selection and Recruitment in Medical Education in AMEE MedEdPublish.

## References

[ref1] Barajaz M Wolfe A Denniston S Kumar S (2018). If You Build It, Will They Come? An Analysis of Candidate Attitudes Toward A New Residency Program. MedEdPublish. Paper No: 24. 10.15694/mep.2018.0000245.1 PMC10711978 38089192

[ref2] Cunliffe J Melton W Hargreaves H Abed R (2018). #ChoosePsychiatry #the60secondchallenge - a thematic analysis of videos published on Twitter about reasons for choosing psychiatry. MedEdPublish. Paper No: 40. 10.15694/mep.2018.0000261.1 PMC10711973 38089197

[ref3] Fink R Kuhn C Taekman J Yanez N (2018). The relationship of Grit to faculty evaluations and standardized test scores of anesthesia residents: a pilot study. MedEdPublish. Paper No: 52. 10.15694/mep.2018.0000273.1

[ref4] Fortin Y Kulasegaram K Kancir J Moineau G Carpentier A (2018). The Direct Economic and Opportunity Costs of the Medical College Admissions Test (MCAT) for Canadian Medical Students. MedEdPublish. Paper No: 22. 10.15694/mep.2018.0000243.1 PMC10711961 38089211

[ref5] García-Estañ J (2018). Studying Medicine and being a doctor in Spain. MedEdPublish. Paper No: 55. 10.15694/mep.2018.0000276.1 PMC10711972 38089186

[ref6] Gardner A Dunkin B (2018). Making progress on identifying those who aren’t making progress: Using situational judgment tests to predict those at risk for remediation and attrition. MedEdPublish. Paper No: 54. 10.15694/mep.2018.0000275.1 PMC10711960 38089233

[ref7] Garrud P Hughes G Greaves S McCracken S Doyle J. (2018) “I’m a Medic” - a web-based, social capital approach to health careers. MedEdPublish. Paper No: 59. 10.15694/mep.2018.0000280.1 PMC10898451 38415014

[ref8] Lee H (2018). Comments on the 2018 Ottawa Consensus Statement - An Admissions Officer’s Point of View. MedEdPublish. Paper No: 49. 10.15694/mep.2018.0000270.1

[ref9] Lombard M Poropat A Alldridge L Rogers G. D. (2018) How can the perceptions and experiences of medical educator stakeholders inform selection into medicine? An interpretative phenomenological pilot study. MedEdPublish. Paper No: 61. 10.15694/mep.2018.0000282.1 PMC10711958 38089195

[ref10] Parsons W McHugh J Yi Y (2018). Traditional Panel Interview versus Multiple Mini-Interview (MMI) in Medical School Admissions: Does Performance differ by Age, Gender, Urban or Rural, or Socioeconomic Status (Findings from one medical school). MedEdPublish. Paper No: 51. 10.15694/mep.2018.0000272.1 PMC10711992 38089204

[ref11] Paton L Tiffin P (2018). Artificial or intelligent? Machine learning and medical selection: possibilities and risks. MedEdPublish. Paper No: 35. 10.15694/mep.2018.0000256.1 PMC10712013 38089244

[ref12] Patterson F Roberts C Hanson M.D Hampe W Eva K (2018). Ottawa Consensus Statement: Selection and Recruitment to the Healthcare Professions. Medical Teacher. 10.1080/0142159X.2018.1498589 30251906

[ref13] Patterson F Hanson M Griffin B (2018). Opening Editorial: Selection and Recruitment in Medical Education. MedEdPublish. Paper No: 1. 10.15694/mep.2018.0000222.1 PMC10704444 38074594

[ref14] Patterson F & Zibarras L . (2011). Exploring the construct of perceived job discrimination and a model of applicant propensity for case initiation in selection. International Journal of Selection & Assessment. 19:3,251–257. 10.1111/j.1468-2389.2011.00553.x

[ref15] Rothstein W Kremers A Goldberg S (2018). Piloting a Tool for Assessment of Person-organization Fit for Residency Applicants: Lessons from Organizational Psychology. MedEdPublish. Paper No: 25. 10.15694/mep.2018.0000246.1

[ref16] Wouters A Croiset G Kusurkar R (2018). Selection and lottery in medical school admissions: who gains and who loses? MedEdPublish. Paper No: 50. 10.15694/mep.2018.0000271.1 PMC10711990 38089206

[ref17] Yingling S Park Y Curry R Monson V Girotti J (2018). Beyond cognitive measures: Empirical evidence supporting holistic medical school admissions practices and professional identity formation. MedEdPublish. Paper No: 53. 10.15694/mep.2018.0000274.1 PMC10711998 38089240

