# Supplementary material for: Closing Editorial: New insights and reflections on the science of selection and recruitment
Source: MedEdPublish (2016). 2018 Dec 18;7:287. [Version 1] doi: 10.15694/mep.2018.0000287.1 (PMC10712004; doi:10.15694/mep.2018.0000287.1)
Supplement: Peer review history

# Reviewer report: Reviewer response for version 1

**DOI:** 10.21956/mep.19738.r29584

**Published:** 2019-01-01

**Authors:** Ronald M Harden

**Affiliations:** 1 AMEE

This review has been migrated. The reviewer awarded 5 stars out of 5 Approaches to selection are on today's agenda in medical education. The 23 papers published in this themed issue make a valuable contribution. This well written closing editorial by Patterson and colleagues does a great job summarising some of the issues raised. Highlighted are some of the complex issues. I recommend this paper to all working in the field of medical education.

Reviewer Expertise:

NA

No decision status is available

# Reviewer report: Reviewer response for version 1

**DOI:** 10.21956/mep.19738.r29585

**Published:** 2018-12-26

**Authors:** John Cookson

**Affiliations:** 1 University of Worcester

This review has been migrated. The reviewer awarded 5 stars out of 5 I also found this helpful as a 'state of the art' review. In particular the indentification of the complex nature of the process and the different stakeholders counsels aganst some of the more simplistic solutions proposed.

Reviewer Expertise:

NA

No decision status is available

# Reviewer report: Reviewer response for version 1

**DOI:** 10.21956/mep.19738.r29582

**Published:** 2018-12-25

**Authors:** Sateesh Babu Arja

**Affiliations:** 1 Avalon University School of Medicine

This review has been migrated. The reviewer awarded 5 stars out of 5 This theme has been interesting one and addressed one of the important and controversial topic in medical education “Selection and recruitment in medical education”. This editorial is very well written, comprehensive, and very informative. The closing editorial touched four important themes new technologies, promoting diversity and widening access, non-academic selection criteria, and postgraduate recruitment. Holistic approach in the selection is very crucucial in the current demanding health professions. Some literature is in favor of traditional interviews, some are in favor of MMIs and some are in favor of situational judgement tests. But it is the institution to develop policies and procedures and to adopt the policy depending on the local and social context.This editorial is very useful for all academic members involved in admission process.

Reviewer Expertise:

NA

No decision status is available

# Reviewer report: Reviewer response for version 1

**DOI:** 10.21956/mep.19738.r29583

**Published:** 2018-12-21

**Authors:** Megan Anakin

**Affiliations:** 1 University of Otago

This review has been migrated. The reviewer awarded 5 stars out of 5 I found the publication of this editorial very timely and helpful as my colleagues and I work together to understand the selection process used in our university’s undergraduate physiotherapy programme. We are wondering if there are steps that can be put in place at the recruitment and selection stages to help address the pressures that are reported by teachers about student diversity, workforce demands, and positive outcomes. This editorial provides a clear overview of many important issues for us to consider. We will be reading the articles in this theme with interest and aim to contribute to this conversation in due course.

Reviewer Expertise:

NA

No decision status is available

# Reviewer report: Reviewer response for version 1

**DOI:** 10.21956/mep.19738.r29586

**Published:** 2018-12-18

**Authors:** Trevor Gibbs

**Affiliations:** 1 AMEE

This review has been migrated. The reviewer awarded 5 stars out of 5 This has been an interesting theme and clearly shows how selection is moving along through keeping up with the changing world. I would hope that this theme can continue as faculty see the emergence of some very important areas of research in this area.Congratulations to the authors in bring this to our attention and collating the results of the various papers. I look forward to reading more papers in this area and would certainly recommend the theme papers to all those involved in the difficult area of selection.

Reviewer Expertise:

NA

No decision status is available
